# Ist die Protonenbestrahlung der Photonenbestrahlung im Hinblick auf das neurokognitive Outcome überlegen?

**DOI:** 10.1007/s00066-020-01692-y

**Published:** 2020-09-30

**Authors:** Semi B. Harrabi

**Affiliations:** Heidelberger Ionenstrahl-Therapiezentrum (HIT), Radioonkologie und Strahlentherapie, Universitätsklinikum Heidelberg, Im Neuenheimer Feld 400, 69120 Heidelberg, Deutschland

## Hintergrund

Die Bestrahlung der kraniospinalen Achse (CSI) ist seit Jahrzehnten ein elementarer Bestandteil bei der Behandlung von Kindern mit Medulloblastom. Je nach Alter des Kindes und Dosierung geht die Behandlung jedoch mit einem erheblichen Risiko für Einbußen der Gedächtnisleistung und negativen Auswirkungen für die weitere psychosoziale Entwicklung einher [1]. Eine risikoadaptierte Reduktion der Bestrahlungsdosis sowie eine Anpassung des Boost-Volumens wirken sich positiv auf die Gedächtnisleistung aus, ohne den onkologischen Behandlungserfolg zu gefährden [2]. Die Protonenbestrahlung kann aufgrund ihrer vorteilhaften physikalischen Eigenschaften im Vergleich zur Photonenbestrahlung die vom gesunden Hirngewebe absorbierte Dosis außerhalb des Zielvolumens weiter reduzieren und somit auch potenziell das Ausmaß der neurokognitiven Einschränkung verringern. Bislang gibt es jedoch keine verlässliche Datenlage, die diesen theoretischen Vorteil eindeutig belegt. Wenngleich nicht in einem prospektiven, randomisierten Setting, so trägt die Arbeit von Kahalley et al. [3] doch wesentlich dazu bei, den Stellenwert der Protonenbestrahlung im Hinblick auf das neurokognitive Outcome zu klären.

## Patienten und Methode

Für diese retrospektive Analyse wurden 79 pädiatrische Patienten des Hospital for the Sick Children (Toronto, Kanada) und des Texas Children’s Hospital (Houston, USA) berücksichtigt, die im Zeitraum von 2007 bis 2018 mit histologisch gesichertem Medulloblastom eine Bestrahlung der Neuroachse plus Boost auf das Tumorbett erhielten. Die Standardtherapie für das US-Kollektiv (*n* = 37) war im Untersuchungszeitraum die Protonenbestrahlung, mangels Verfügbarkeit der Protonentechnik wurden alle kanadischen Patienten (*n* = 42) mit Photonen bestrahlt. Die Bestrahlung der Neuroachse erfolgte risikoadaptiert im Rahmen aktueller multimodaler Therapiekonzepte (SJMB03/ClinicalTrials.gov Identifier: NCT00085202 oder SJMB12/ClinicalTrials.gov Identifier: NCT01878617) nach Resektion entweder dosisreduziert mit 15–23,4 Gy oder regulär mit 30,6–39,6 Gy. Das Boost-Volumen umfasste bei keinem Patienten die gesamte hintere Schädelgrube, es wurde vielmehr nur das Tumorbett mit einem CTV-Saum von 5 bis 10 mm bis zu einer kumulativen Gesamtdosis von 51,0 bis 59,4 Gy behandelt. Zwar war an beiden Zentren eine prospektive neuropsychologische Betreuung und Testung Standard, jedoch unterschieden sich die zum Einsatz kommenden Testbatterien. Neben einem globalen Assessment des IQ wurde v. a. auch Augenmerk auf einzelne Subdomänen wie etwa Sprachverständnis, wahrnehmungsgebundenes logisches Denken, Arbeitsgedächtnis und Verarbeitungsgeschwindigkeit gelegt.

## Ergebnisse

Es wurden die longitudinalen Ergebnisse der neurokognitiven Testungen von 79 Kindern untersucht, davon 37 nach Protonenbestrahlung und 42 nach Photonenbestrahlung. Das mediane Follow-up betrug 4,3 Jahre. Im Hinblick auf ihre klinischen und demografischen Charakteristika ist hervorzuheben, dass sich die beiden Kohorten sowohl bei der Verschreibungsdosis für den Boost als auch beim Sicherheitssaum für den Boost signifikant unterschieden. Während bei der Photonenbestrahlung ausnahmslos ein CTV-Saum von 10 mm verwendet wurde, waren es bei 37,8 % der Patienten in der Protonengruppe lediglich 5 mm. Die mediane Boost-Dosis war mit 55,8 Gy im Photonenarm höher als im Protonenarm (54 Gy). Die weiteren Charakteristika, wie etwa Geschlecht, Alter oder Dosis der Neuroachse, zeigten keine relevanten Unterschiede.

Kinder, die mit Protonen behandelt wurden, zeigten im Verlauf keine Veränderung des globalen IQs im Vergleich zur Baseline. Im Gegensatz dazu wurde bei der Photonenkohorte ein statistisch signifikanter Verlust von durchschnittlich 0,9 IQ-Punkten pro Jahr festgestellt.

Bei der Untersuchung des Sprachverständnisses zeigte sich in beiden Gruppen weder zur Baseline noch im weiteren Verlauf eine signifikante Veränderung.

Das wahrnehmungsgebundene logische Denken hingegen verbesserte sich sogar nach Protonenbestrahlung um durchschnittlich einen Punkt pro Jahr, während nach Photonenbestrahlung tendenziell eine Verschlechterung von durchschnittlich 0,8 Punkten pro Jahr beobachtet wurde, jedoch ohne statistische Signifikanz zu erreichen.

Bei der Analyse des Arbeitsgedächtnisses blieben die Ergebnisse während des Nachbeobachtungszeitraums für Protonen unverändert zum Ausgangsbefund, wohingegen nach Photonenbestrahlung eine signifikante Verschlechterung von 2,2 Punkten pro Jahr festgestellt wurde.

Im Gegensatz zu den anderen Subdomänen zeigte sich die Verarbeitungsgeschwindigkeit in beiden Behandlungsgruppen gleichermaßen beeinträchtigt und nahm im Untersuchungszeitraum um durchschnittlich 0,9 Punkte pro Jahr ab.

Sämtliche Tests wurden auch separat für die SJMB03-Kohorte (*n* = 65) ausgewertet. Im Vergleich zum Gesamtkollektiv zeigte sich bei nunmehr gleichen Rahmenbedingungen (Chemotherapie, Boost-Dosis und CTV-Saum) ein ähnliches Ergebnis: Vorteile für die Protonenbestrahlung bei Arbeitsgedächtnis und wahrnehmungsgebundenem logischem Denken und keine Veränderung bei der Verarbeitungsgeschwindigkeit in beiden Gruppen. Im Gegensatz zur kombinierten Auswertung wurden in der Protonengruppe im Verlauf signifikant bessere Scores für das Sprachverständnis erzielt, jedoch ging der zuvor statistisch relevante Vorteil für den globalen IQ verloren.

## Interpretation der Autoren

Die Auswirkungen einer protonen- oder photonenbasierten Behandlung von pädiatrischen Patienten mit Medulloblastom auf das neurokognitive Outcome wurden erstmals mit vergleichbaren und aus demselben Behandlungszeitraum stammenden Protokollen untersucht. Die Bestrahlung mit Protonen führt im Vergleich zur Behandlung mit Photonen zu einem vorteilhaften Ergebnis in den meisten untersuchten Domänen. Lediglich die Verarbeitungsgeschwindigkeit zeigte sich unabhängig von der zum Einsatz kommenden Strahlenart beeinträchtigt. Die Ergebnisse untermauern die Rationale, Protonen bei der Behandlung von Hirntumoren mit dem Ziel der Bewahrung der Gedächtnisleistung anzuwenden.

## Kommentar

In den letzten Jahren konnte sich die Protonenbestrahlung insbesondere bei der Behandlung von Hirntumoren im Kindesalter als Therapieoption der ersten Wahl etablieren. Trotz einer international rasant ansteigenden Anzahl neuer Einrichtungen stellt eine mangelnde Verfügbarkeit oftmals die Hauptursache dar, wenn die Behandlung dennoch mit Photonen durchgeführt wird. Dank zahlreicher dosimetrischer Vergleichsstudien ist die überlegene Dosisverteilung der Protonenbestrahlung unstrittig [4–6]. Ob sich jedoch die konformalere Behandlung und die geringere Niedrigdosis im umliegenden gesunden Gewebe tatsächlich in klinisch relevante Vorteile übersetzen, konnte noch nicht für alle postulierten Indikationen zweifelsfrei belegt werden und wird z. T. nach wie vor kontrovers diskutiert. Dies ist aber von essenzieller Bedeutung, denn dank stetiger Verbesserungen bei Diagnostik, Therapie und Nachsorge besiegen in Deutschland über 80 % der pädiatrischen Patienten ihre onkologische Erkrankung und werden zu Langzeitüberlebenden [7]; 1975 waren es nur knapp 50 %. Die Vermeidung schwerwiegender therapiebedingter Spätfolgen rückt daher neben der schieren Kontrolle der Grunderkrankung zunehmend in den Fokus. Bekanntermaßen ist die Bürde der Therapie nach einer kraniospinalen Bestrahlung in jungen Jahren besonders hoch und kann u. a. zu erheblichen körperlichen und neurokognitiven Einschränkungen führen, die ein Leben in Selbstständigkeit erschweren. Die Rationale zur lokalen Bestrahlung von Tumoren des zentralen Nervensystems oder der Schädelbasis mittels Protonen ist deshalb weithin akzeptiert, existieren doch für viele klinisch relevante Endpunkte klare Dosis-Wirkungs-Beziehungen. So haben beispielsweise Vatner et al. sehr deutlich das steigende Risiko endokriner Ausfälle mit steigender Dosis am Hypothalamus aufgezeigt [8]. Im Umkehrschluss kann der „benefit“ der geringeren Dosisbelastung des Hypothalamus oder der Hypophyse durch die Verwendung von Protonen bei vorliegender Vergleichsplanung mit Photonen abgeschätzt werden.

Deutlich schwieriger gestaltet sich der eindeutige Beleg für ein verbessertes neurokognitives Outcome nach Protonenbestrahlung. Prospektive, randomisierte Evidenz sucht man vergeblich. Es finden sich jedoch vermehrt Arbeiten, die die Ergebnisse neuropsychologischer Testungen beider Bestrahlungsmodalitäten vergleichend gegenüberstellen. So konnten Kahalley et al. bereits in einer Vorarbeit feststellen, dass es nach Protonenbestrahlung zu keiner Verschlechterung des IQs kam, jedoch erreichte die Abnahme von 1,1 Punkten pro Jahr nach photonenbasierter Bestrahlung keine statistische Signifikanz [9]. Auch Gross et al. fanden heraus, dass sich eine Protonenbestrahlung bei der Behandlung kindlicher Hirntumoren im Vergleich zur konventionellen Bestrahlung positiv auf das neuropsychologische Outcome auswirkt [10]. Wenngleich zunehmend Evidenz für eine gleichbleibende neurokognitive Leistungsfähigkeit nach Protonenbestrahlung geschaffen wird [11, 12], muss jedoch kritisch bemerkt werden, dass die Aussagekraft der Ergebnisse zum Teil limitiert ist, da sie sich entweder auf einen historischen Vergleich mit Photonen beziehen oder möglicherweise einem Selektionsbias unterliegen, da der sozioökonomische Status im Protonenkollektiv deutlich erhöht war. Bekanntermaßen korreliert dieser mit besseren Testergebnissen und führt u. a. aufgrund einer erhöhten Möglichkeit zur Mobilität zu einer deutlich erhöhten Wahrscheinlichkeit, eine Protonenbestrahlung zu erhalten [13]. Die Kahalley-Arbeit überwindet eben diese Limitationen, da der Vergleich zu fairen Bedingungen stattfand: Es kamen moderne Photonenverfahren mit den gleichen (zeitgenössischen) Protokollvorgaben im Hinblick auf Zielvolumendefinition und Dosierung bei einem im Hinblick auf demografische, klinische oder sozioökonomische Charakteristika vergleichbaren Kollektiv zum Einsatz.

Besonders bemerkenswert ist, dass der positive Einfluss nicht an einem Kollektiv mit rein fokaler Bestrahlung gezeigt wurde, sondern vielmehr bei Patienten mit kraniospinaler Bestrahlung. Die bisherige Annahme, dass die gefürchteten Einbußen der Gedächtnisleistung v. a. dem Dosisbeitrag durch die Ganzhirnbestrahlung geschuldet sind, muss kritisch überdacht werden. Mitursächlich ist sicherlich die risikoadaptierte Dosisreduktion bei Bestrahlung der kraniospinalen Achse. Ebenso relevant dürfte aber auch die Anpassung des Zielvolumens bei der Boost-Bestrahlung hin zur Tumorbettbestrahlung anstelle der gesamten hinteren Schädelgrube sein. Wie es scheint, ist aber nicht nur die zerebelläre Integraldosis, sondern vielmehr auch die vom supratentoriellen Gehirn absorbierte Dosis ausschlaggebend für das funktionale Ergebnis. Und genau da kann die Protonenbestrahlung dank ihrer vorteilhaften physikalischen Eigenschaften zu einer relevanten Reduktion und damit einer geringeren Beeinträchtigung der Gedächtnisleistung beitragen.

## Fazit

Allen bekannten Limitationen retrospektiver Analysen zum Trotz liefert die Arbeit von Kahalley et al. die Evidenz für ein besseres neurokognitives Outcome bei den kindlichen Patienten nach Protonenbestrahlung – insbesondere auch im Vergleich mit der modernen photonenbasierten Bestrahlung. Zwar ist damit der Vorteil der Protonenbestrahlung im Hinblick auf das neurokognitive Outcome nicht abschließend belegt, aber wie bei einem Mosaik wird das Gesamtbild dank dieses weiteren wichtigen Puzzlestücks deutlicher erkennbar.

*Semi Harrabi, Heidelberg*
